# Degradation of Methyl 2-Aminobenzoate (Methyl Anthranilate) by H_2_O_2_/UV: Effect of Inorganic Anions and Derived Radicals

**DOI:** 10.3390/molecules22040619

**Published:** 2017-04-12

**Authors:** Grazia Maria Lanzafame, Mohamed Sarakha, Debora Fabbri, Davide Vione

**Affiliations:** 1 Institut de Chimie de Clermont-Ferrand, Clermont Université, Université Blaise Pascal, F-63177 Aubière, France; grazialanzafame@gmail.com (G.M.L.); mohamed.sarakha@uca.fr (M.S.); 2 Dipartimento di Chimica, Università di Torino, Via Pietro Giuria 5, 10125 Torino, Italy; debora.fabbri@unito.it; 3 Centro Interdipartimentale NatRisk, Università di Torino, Largo Paolo Braccini 2, 10095 Grugliasco (TO), Italy

**Keywords:** advanced oxidation processes, methyl anthranilate, methyl 2-aminobenzoate, hydrogen peroxide, photodegradation intermediates, emerging contaminants

## Abstract

This study shows that methyl 2-aminobenzoate (also known as methyl anthranilate, hereafter MA) undergoes direct photolysis under UVC and UVB irradiation and that its photodegradation is further accelerated in the presence of H_2_O_2_. Hydrogen peroxide acts as a source of hydroxyl radicals (·OH) under photochemical conditions and yields MA hydroxyderivatives. The trend of MA photodegradation rate vs. H_2_O_2_ concentration reaches a plateau because of the combined effects of H_2_O_2_ absorption saturation and ·OH scavenging by H_2_O_2_. The addition of chloride ions causes scavenging of ·OH, yielding Cl_2_·^−^ as the most likely reactive species, and it increases the MA photodegradation rate at high H_2_O_2_ concentration values. The reaction between Cl_2_·^−^ and MA, which has second-order rate constant $k_{Cl_{2}^{•-}+MA}$ = (4.0 ± 0.3) × 10^8^ M^−1^·s^−1^ (determined by laser flash photolysis), appears to be more selective than the ·OH process in the presence of H_2_O_2_, because Cl_2_·^−^ undergoes more limited scavenging by H_2_O_2_ compared to ·OH. While the addition of carbonate causes ·OH scavenging to produce CO_3_·^−^ ($k_{CO_{3}^{•-}+MA}$ = (3.1 ± 0.2) × 10^8^ M^−1^·s^−1^), carbonate considerably inhibits the photodegradation of MA. A possible explanation is that the elevated pH values of the carbonate solutions make H_2_O_2_ to partially occur as HO_2_^−^, which reacts very quickly with either ·OH or CO_3_·^−^ to produce O_2_·^−^. The superoxide anion could reduce partially oxidised MA back to the initial substrate, with consequent inhibition of MA photodegradation. Fast MA photodegradation is also observed in the presence of persulphate/UV, which yields SO_4_·^−^ that reacts effectively with MA ($k_{SO_{4}^{•-}+MA}$ = (5.6 ± 0.4) × 10^9^ M^−1^·s^−1^). Irradiated H_2_O_2_ is effective in photodegrading MA, but the resulting MA hydroxyderivatives are predicted to be about as toxic as the parent compound for aquatic organisms (most notably, fish and crustaceans).

## 1. Introduction

Methyl 2-aminobenzoate (MA, C_8_H_9_NO_2_) is a clear liquid that occurs in many essential oils. It has a melting point of 24 °C, a boiling point of 256 °C, and a density of 1.17 g·mL^−1^ [1]. MA can be found in Concord grapes, jasmine, bergamot, lemon, orange, and strawberries, and it is used as bird repellent in the protection of food crops such as corn, sunflower, rice, and fruits [2]. MA is also employed to prevent birds from accessing oil spill–contaminated water or water pools near airports, in the latter case reducing the risk of collision with aircraft [3]. MA is used as well for the flavouring of candies, soft drinks, chewing gum, and medicines [4], which suggests that it has limited toxicity towards mammals, including humans. Its LD50 (lethal dose 50) values for rats and rabbits are in fact in the g/(Kg body weight) range for either oral or dermal uptake [5]. Despite these apparently favourable features, MA shows non-negligible toxicity for aquatic organisms, with LC50 (lethal concentration 50 in water, i.e., acute toxicity) values of 20–30 mg·L^−1^ for fish [6]. More importantly, it can cause chronic effects to both fish and crustaceans at 10–70 µg·L^−1^ levels [7,8,9]. Therefore, toxicity to fish is to be taken into account when using MA to protect aquaculture facilities from predation by birds [10]. MA is also poorly biodegradable, little volatile, and it undergoes limited partitioning to solids. Moreover, its predicted hydrolysis time is in the range of several months to some years [11]. Following ingestion as a food additive and excretion, this compound is unlikely to be eliminated from the aqueous phase of the wastewater treatment plants (estimates for MA elimination are in the range of a few percent, mostly accounted for by sludge adsorption) [11]. Therefore, MA could easily reach surface water environments. Unfortunately, concentration data of MA in surface waters are extremely difficult to be found in the literature, as are data concerning the elimination of MA from aqueous solutions including wastewater.

Poor elimination during wastewater treatment is a widespread feature of several emerging substances used as drugs, fragrances, fire extinguishers etc. These compounds can be found in surface waters at significant levels due to point-source emission at the treatment plant outlets [12,13]. A likely future development in wastewater treatment will be the update of the existing plants to enable the removal of emerging contaminants. As technological upgrade options, Advanced Oxidation Processes (AOPs) are among the best promising tools [14,15]. Most AOPs are based on the thermal, electrochemical, photochemical or sonochemical generation of hydroxyl radicals (·OH), in the homogeneous phase or in heterogeneous systems (e.g., heterogeneous photocatalysis, [16]). The ·OH radicals are very strong oxidants and they can react with a very wide variety of organic and inorganic molecules, including pollutants. The relevant reactions include electron or hydrogen transfer, as well as addition to double bonds and aromatic rings [17]. Among AOPs, the UV irradiation of hydrogen peroxide (hereafter, H_2_O_2_/UV) presents several advantages including the elevated quantum yield of ·OH photogeneration (the reaction H_2_O_2_ + hν → 2 ·OH has $\Phi_{H_{2}O_{2}}$ ~ 0.5 and ΦOH• ~ 1 [17,18]), the low cost of H_2_O_2_, and the formation of rather innocuous reaction by-products from H_2_O_2_ itself (mostly H_2_O and O_2_) [19,20]. H_2_O_2_ is also water-miscible and it is relatively safe to store and transport pending some precautions, but its water solutions need to be added with stabilisers (e.g., stannate, pyrophosphate, nitrate, colloidal silicate) that will finally end up in the water undergoing treatment [18]. Moreover, toxic or otherwise harmful photodegradation intermediates may be formed when dealing with the treatment of certain organic pollutants [21]. Another issue is that ·OH reacts not only with the target contaminant, but also with natural organic matter (NOM) and with inorganic anions that occur in aqueous solution. While NOM is mostly a ·OH quencher, in the case of some anions the framework is more complicated because their oxidation by ·OH yields reactive radical species, which are less reactive than ·OH itself but could still initiate some degradation processes on their own. Examples are the ·OH-induced formation of the carbonate radical (CO_3_·^−^) from carbonate and bicarbonate, of dichloride (Cl_2_·^−^) from chloride, of dibromide (Br_2_·^−^) from bromide, and of nitrogen dioxide (·NO_2_) from nitrite [22]. Moreover, one of the possibilities to reduce the consumption of reactive species by the organic and inorganic components of natural waters is to replace ·OH with the more selective sulphate radical, SO_4_·^−^, which reacts with several pollutants but undergoes less interference from NOM and inorganic anions compared to ·OH [23]. To produce SO_4_·^−^, it is often sufficient to replace H_2_O_2_ with the analogous peroxide persulphate (S_2_O_8_^2−^) in comparable processes [23,24].

The goal of this work is to study the photoinduced degradation of MA under UV irradiation (direct UVC photolysis, here used as benchmark) and with H_2_O_2_/UV and persulphate/UV treatments, as well as to assess the effect on the process of common inorganic anions such as chloride and carbonate. To better assess the effect of the added anions, the reactivity of CO_3_·^−^ and Cl_2_·^−^ with MA was studied by using the nanosecond laser flash photolysis technique. Because MA is not harmless to aquatic environments, this study investigates the following: (i) whether and to what extent MA could be photodegraded under AOP conditions, also in the presence of inorganic anions such as chloride and carbonate; and (ii) the potential of MA photodegradation to produce intermediates that might have higher impact than the parent compound, and that could be formed during the AOP removal of MA and/or other contaminants.

## 2. Results and Discussion

### 2.1. MA Photodegradation by UV and H_2_O_2_/UV

**Figure 1:**
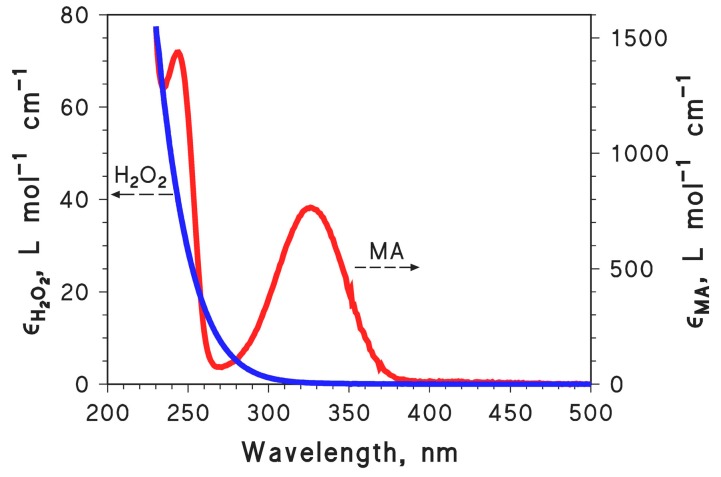
Absorption spectra (molar absorption coefficients) of methyl 2-aminobenzoate (MA) (right Y-axis) and H_2_O_2_ (left Y-axis).

**Figure 2:**
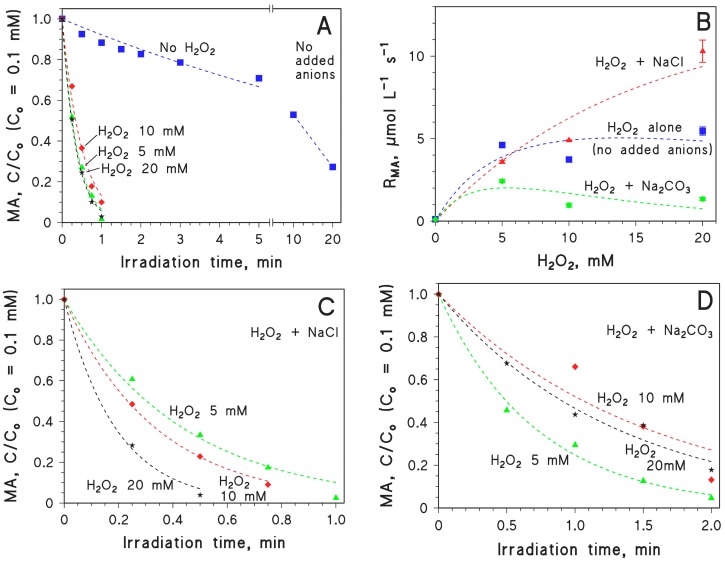
(**A**) Time trend of 0.1 mM MA under UVC irradiation, alone or in the presence of different concentration values of H_2_O_2_ (varied in the range of 0–20 mM); (**B**) Initial photodegradation rates of 0.1 mM MA (*R*_MA_) as a function of H_2_O_2_ concentration, alone or in the presence of 0.1 M NaCl or 0.1 M Na_2_CO_3_; (**C**) Time trend of 0.1 mM MA in the presence of 0.1 M NaCl and different concentration values of H_2_O_2_ (varied in the range of 5–20 mM); (**D**) Time trend of 0.1 mM MA in the presence of 0.1 M Na_2_CO_3_ and different concentration values of H_2_O_2_ (varied in the range of 5–20 mM). The pH values of the studied systems were ~neutral, with the exception of the systems containing Na_2_CO_3_. The error bars shown in panel (**B**) represent the uncertainty associated to the calculation of the photodegradation rates by fitting the MA time trend data with a pseudo-first order kinetic model (intra-series variability). In several cases the error bars were smaller than the data points. The reproducibility between experimental replicas (inter-series variability) was in the range of 15–20%.

The photoinduced degradation of 0.1 mM MA was first studied under UVC irradiation alone (lamp maximum emission at 254 nm) and under UVC irradiation in the presence of different concentration values of H_2_O_2_ (see Figure 1 for the absorption spectra of MA and H_2_O_2_). The MA time evolution under these conditions is reported in Figure 2A, while Figure 2B reports the trend of the photodegradation rate of MA (*R*_MA_) as a function of the H_2_O_2_ concentration. Table 1 reports the pseudo-first order photodegradation rate constants of MA (*k*_MA_) for this and other series of experiments. Note that *R*_MA_ = *k*_MA_ [MA]_o_, where [MA]_o_ = 0.1 mM is the initial concentration of MA.

Some MA photodegradation with a half-life time of approximately 10 min took place in the absence of H_2_O_2_, due to MA direct photolysis. The direct photolysis quantum yield of MA was calculated as follows [25]:

(1) $$\Phi_{MA}=\frac{R_{MA}}{\int_{\lambda }p{}^{\circ}(\lambda )\;(1-10^{A_{MA}(\lambda )})\;d\lambda }$$

where p°(λ) is the incident spectral photon flux density of the lamp and $A_{MA}(\lambda )$ is the absorbance of 0.1 mM MA. The photolysis quantum yield was measured in separate experiments under monochromatic irradiation (at 254 and 325 nm) and under broadband irradiation (see Supplementary Material for the detailed results). The $\Phi_{MA}$ values obtained in the different conditions are in the order of magnitude of 10^−3^. The value at 254 nm ($\Phi_{MA}^{254nm}$ = 3.8 × 10^−3^) is the most relevant to our steady irradiation experiments, and a decrease was observed in the values of $\Phi_{MA}$ as the irradiation wavelength increased. Therefore, when applying artificial irradiation, the UVC spectral range and in particular the radiation at 254 nm (very near the UVC absorption maximum of MA, see Figure 1) appears to be the most suitable option to induce MA direct photolysis.

The addition of H_2_O_2_ considerably accelerated the photodegradation of MA, and relatively similar kinetics were obtained in the H_2_O_2_ concentration range of 5–20 mM. A plateau trend of *R*_MA_ vs. [H_2_O_2_] is apparent in Figure 2B, and in principle it might be accounted for by two different phenomena: (i) saturation of H_2_O_2_ absorption with increasing H_2_O_2_ concentration; and (ii) offset between photoinduced ·OH generation, and ·OH scavenging by H_2_O_2_ itself. The first effect depends on the absorbance of H_2_O_2_. Considering $\varepsilon_{H_{2}O_{2},254nm}$ ~ 15 L·mol^−1^·cm^−1^ and assuming *b* = 2 cm as the optical path length inside the irradiated solutions, the absorbance of the studied H_2_O_2_ solutions was approximately 0.15 (5 mM H_2_O_2_), 0.3 (10 mM), and 0.6 (20 mM). The absorbance of 0.1 mM MA at 254 nm is $A_{MA,254nm}$ ~ 0.2, and the fraction of radiation absorbed by H_2_O_2_ in the irradiated systems can be calculated as follows [25]:

(2) $${℘}_{H_{2}O_{2},254nm}^{H_{2}O_{2}+MA}=\frac{\varepsilon_{H_{2}O_{2},254nm}\;b\;[H_{2}O_{2}]}{\varepsilon_{H_{2}O_{2},254nm}\;b\;[H_{2}O_{2}]+A_{MA,254nm}}\;\left[\;1-10^{-\left(\varepsilon_{H_{2}O_{2},254nm}\;b\;[H_{2}O_{2}]+A_{MA,254nm}\right)}\right]$$

On this basis, the values of ${℘}_{H_{2}O_{2},254nm}^{H_{2}O_{2}+MA}$ as a function of the H_2_O_2_ concentration are 0.24 (H_2_O_2_ 5 mM), 0.41 (H_2_O_2_ 10 mM), and 0.63 (H_2_O_2_ 20 mM). Because the lamp radiation can be considered as monochromatic as a first approximation, these values are directly proportional to the formation rate of ·OH produced by the irradiation of H_2_O_2_ (ROH•H2O2+hν ∝ ${℘}_{H_{2}O_{2},254nm}^{H_{2}O_{2}+MA}$). The direct proportionality constant between ROH•H2O2+hν and ${℘}_{H_{2}O_{2},254nm}^{H_{2}O_{2}+MA}$ includes the formation quantum yield of ·OH by irradiated H_2_O_2_ and the incident photon flux in solution, which are constant values that can be included into a proportionality parameter α (as ROH•H2O2+hν=α ℘H2O2,254nmH2O2+MA). The photogenerated ·OH can react with either MA or H_2_O_2_, and in the latter case the second-order reaction rate constant is kOH+H2O2• = 2.7 × 10^7^ M^−1^·s^−1^ [26]. By assuming kOH+MA• as the (unknown) second-order reaction rate constant between ·OH and MA, the competition kinetics between MA and H_2_O_2_ yields the following results for the MA photodegradation rate ($R_{MA}$):

(3) RMA=ROH•H2O2+hν kOH+MA• [MA]kOH+MA• [MA]+kOH+H2O2•[H2O2]=α ℘H2O2,254nmH2O2+MA 11+ kOH+H2O2•kOH+MA• [H2O2][MA]

With the known values of $\varepsilon_{H_{2}O_{2},254nm}$ ~ 15 M^−1^·cm^−1^, $A_{MA,254nm}$ ~ 0.2, [MA] = 0.1 mM and kOH+H2O2• = 2.7 × 10^7^ M^−1^·s^−1^, it was possible to fit reasonably well the $R_{MA}$ vs. [H_2_O_2_] experimental data reported in Figure 2B (see dashed curve in the figure). The fit results suggested that kOH+MA• would be about two orders of magnitude higher than kOH+H2O2•. This means that the reaction of ·OH with H_2_O_2_ is expected to prevail over that with 0.1 mM MA for [H_2_O_2_] > 10 mM, which is right within the investigated range of H_2_O_2_ concentrations.

### 2.2. Effect of Inorganic Anions on MA Photodegradation

The effect of anions commonly occurring in surface waters, and most notably of chloride and carbonate, on the photodegradation of MA induced by H_2_O_2_/UV was studied upon UVC irradiation of MA, H_2_O_2_, and, where relevant, NaCl or Na_2_CO_3_.

The time evolution of 0.1 mM MA in the presence of 0.1 M NaCl and different concentration values of H_2_O_2_ is reported in Figure 2C, and the corresponding photodegradation rates are reported in Figure 2B. The figure shows that MA photodegradation became progressively faster as the H_2_O_2_ concentration increased and, differently from the previous case (MA + H_2_O_2_ + UV, without chloride), there was no obvious plateau trend. The experimental rate data could be fitted well with an equation of the form $R_{MA}=\beta \;{℘}_{H_{2}O_{2},254nm}^{H_{2}O_{2}+MA}$, where β is a constant proportionality factor (see the dashed curve in Figure 2B). In this case it seems that the observed trend just mirrored the photon absorption by H_2_O_2_, with no need to invoke an additional competition kinetics between MA, H_2_O_2_ and the reactive transient species. Moreover, at elevated H_2_O_2_ concentration the photodegradation of MA was considerably faster in the presence of 0.1 M NaCl than in the absence of chloride. These pieces of evidence suggest that the prevailing reactive species in the MA/H_2_O_2_/Cl^−^/UV system is very unlikely to be ·OH, which is expected to produce a plateau trend as per the above discussion. A different transient species should rather be involved, inducing competition kinetics between MA and H_2_O_2_ to a far lesser extent than ·OH. This reactive transient, provisionally indicated here as X, should react with MA and H_2_O_2_ in such a way that kX+H2O2(kX+MA)−1 « kOH+H2O2•(kOH+MA•)−1. If this condition is met, one has $\frac{k_{X+H_{2}O_{2}}}{k_{X+MA}\;[MA]}\;[H_{2}O_{2}]$ < 1 in Equation (4), which differs from Equation (3) in that the ·OH-based terms are replaced by X-based ones:

(4) $$R_{MA}=R_{X}^{H_{2}O_{2}+h\nu }\;\frac{1}{1+\;\frac{k_{X+H_{2}O_{2}}}{k_{X+MA}\;[MA]}\;[H_{2}O_{2}]}$$

In the presence of ·OH + Cl^−^, the following reactions may take place [26,27,28]:

·OH + Cl^−^ ⇆ HOCl·^−^   **[*K*_eq,5_ = 0.70 M^−1^]** (5)

HOCl^−^· + H^+^ ⇆ H_2_O + Cl·   **[*K*_eq,6_ = 1.6×10^7^ M^−^^1^]** (6)

Cl· + Cl^−^ ⇆ Cl_2_·^−^       **[*K*_eq,7_ = 1.9×10^5^ M^−1^]** (7)

Based on the above reactions, potential X-species in the system are HOCl·^−^, Cl·, and Cl_2_·^−^. The reactivity of Cl_2_·^−^ can be studied by laser flash photolysis, thus one can check the possible involvement of Cl_2_·^−^ in MA photodegradation by measuring $k_{Cl_{2}^{•-}+MA}$.

In the H_2_O_2_/Na_2_CO_3_/UV system with 0.1 M Na_2_CO_3_, the photodegradation of MA did not accelerate when increasing [H_2_O_2_] above 5 mM (see Figure 2D for the MA time trends, and Figure 2B for the corresponding photodegradation rates). The ·OH reactions with carbonate and bicarbonate are more straightforward than in the case of chloride and they lead to the unequivocal formation of CO_3_·^−^ as additional reactive species [26,29]:

·OH + HCO_3_^−^ → H_2_O + CO_3_·^−^ (8)

·OH + CO_3_^2−^ → OH^−^ + CO_3_·^−^ (9)

A comparison of the MA photodegradation rates in the systems “H_2_O_2_ alone” and “H_2_O_2_ + Na_2_CO_3_” in Figure 2B shows that the rates were lower in the presence of carbonate, coherently with the replacement of ·OH with the less reactive species CO_3_·^−^. Moreover, the ratio $R_{MA}^{H_{2}O_{2}\;alone}{\left(R_{MA}^{H_{2}O_{2}\;+\;Na_{2}CO_{3}}\right)}^{-1}$ increased with increasing [H_2_O_2_]. A potential explanation for this phenomenon is that H_2_O_2_ competes more effectively with MA, for reaction with CO_3_·^−^, than for reaction with ·OH. In other words, this hypothesis leads to the assumption that kCO3•−+H2O2(kCO3•−+MA)−1>kOH+H2O2•(kOH+MA•)−1. Considering that $k_{CO_{3}^{•-}+H_{2}O_{2}}$ = 8 × 10^5^ M^−1^·s^−1^ is known from the literature [30], the measurement of $k_{CO_{3}^{•-}+MA}$ by laser flash photolysis is an appropriate test for this hypothesis.

### 2.3. MA Photodegradation by Persulphate/UV

The UV irradiation of persulphate yields the sulphate radical, SO_4_·^−^ [31,32,33]. This radical has similar if not higher reduction potential compared to ·OH, but it tends to be preferentially involved in charge-transfer reactions while ·OH often triggers hydrogen-transfer or addition processes in comparable conditions [17,34].

S_2_O_8_^2−^ + hν → 2 SO_4_·^−^ (10)

**Figure 3:**
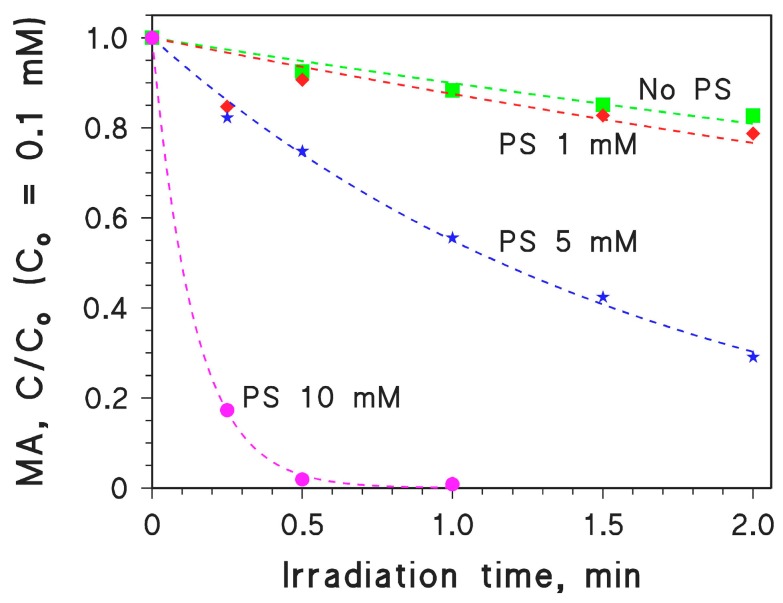
Time trend of 0.1 mM MA upon UVC irradiation, alone or in the presence of different concentration values of Na_2_S_2_O_8_ (persulphate, PS). The PS concentration was varied in the range of 0–10 mM.

The time trend of 0.1 mM MA upon UVC irradiation in the presence of varying concentration values of sodium persulphate (PS) is reported in Figure 3. The figure shows that PS above 1 mM concentration could considerably accelerate the photodegradation of MA, and that the photodegradation became considerably faster as the PS concentration was higher. Moreover, while there was limited difference between the MA time trends with 5 or 10 mM H_2_O_2_, the photodegradation of MA with 10 mM PS was considerably faster compared to 5 mM PS. This result suggests that the reaction between SO_4_·^−^ and PS interferes with MA photodegradation to a lesser extent than the reaction between ·OH and H_2_O_2_.

### 2.4. Second-Order Reaction Rate Constants of MA with Cl_2_·^−^, CO_3_·^−^ and SO_4_·^−^

**Figure 4:**
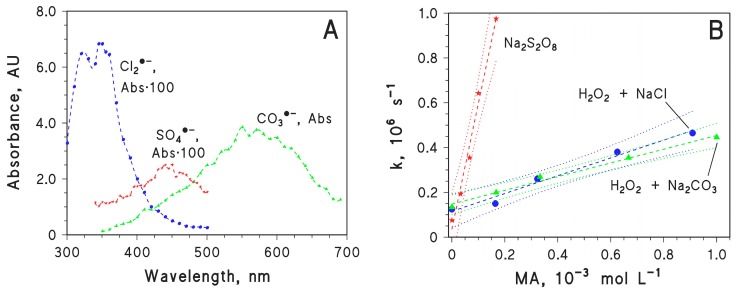
(**A**) Absorption spectra of the radicals CO_3_·^−^, Cl_2_·^−^, and SO_4_·^−^ produced by laser flash photolysis of 2.5 mM H_2_O_2_ + 0.1 M Na_2_CO_3_ (CO_3_·^−^), of 2.5 mM H_2_O_2_ + 0.01 M NaCl (pH 3, adjusted with HClO_4_) (Cl_2_·^−^), and of 10 mM Na_2_S_2_O_8_ (SO_4_·^−^). The absorbance signals were taken soon after the laser pulse. Laser irradiation at 266 nm, 35 mJ·pulse^−1^; (**B**) First-order decay constants of the studied radical species (SO_4_·^−^, Cl_2_·^−^, CO_3_·^−^) as a function of MA concentration. The radical species were obtained by laser irradiation of 10 mM Na_2_S_2_O_8_ (SO_4_·^−^), of 2.5 mM H_2_O_2_ + 0.01 M NaCl at pH 3 (Cl_2_·^−^), and of 2.5 mM H_2_O_2_ + 0.1 M Na_2_CO_3_ (CO_3_·^−^). The slopes of the lines give the second-order reaction rate constants between the relevant radicals and MA (Stern-Volmer approach).

The second-order reaction rate constants between MA and three reactive transient species (Cl_2_·^−^, CO_3_·^−^, and SO_4_·^−^) were measured by means of the laser flash photolysis technique. The radical Cl_2_·^−^ was produced by laser irradiation of H_2_O_2_ + NaCl (0.01 M chloride) at pH 3 by HClO_4_, under which conditions the equilibria of reactions (4–6) are shifted towards the products and there is a consequent enhancement of the formation of Cl_2_·^−^ [26,27,28]. As far as the other transient species are concerned, CO_3_·^−^ was produced by laser irradiation of H_2_O_2_ + Na_2_CO_3_, and SO_4_·^−^ was produced by laser irradiation of Na_2_S_2_O_8_. The actual occurrence of these radicals as the main transient species in the laser-irradiated solutions has been demonstrated in previous studies [35,36]. Figure 4A reports the absorption spectra of the studied solutions undergoing laser flash photolysis, obtained just after the laser pulse. Based on these results, in successive experiments the radical Cl_2_·^−^ was monitored at 350 nm, CO_3_·^−^ at 550 nm, and SO_4_·^−^ at 450 nm. Figure 4B reports the trends of the pseudo-first order decay constants *k* of each transient as a function of the MA concentration. Following the Stern-Volmer approach, the slopes of *k* vs. [MA] represent the second-order reaction rate constants of the transient species with MA. We obtained $k_{Cl_{2}^{•-}+MA}$ = (4.0 ± 0.3) × 10^8^ M^−1^·s^−1^ (the error bounds represent the σ-level uncertainty), $k_{CO_{3}^{•-}+MA}$ = (3.1 ± 0.2) × 10^8^ M^−1^·s^−1^, and $k_{SO_{4}^{•-}+MA}$ = (5.6 ± 0.4) × 10^9^ M^−1^·s^−1^. These values are consistent with the typical reactivity of the three transient species [30]. 

The formation of CO_3_·^−^ and SO_4_·^−^ upon either laser-based or steady-state irradiation of, respectively, H_2_O_2_ + Na_2_CO_3_ and Na_2_S_2_O_8_ is rather straightforward [35,36]. In the case of H_2_O_2_ + NaCl, the laser irradiation took place at pH 3 to ensure the formation of Cl_2_·^−^. In contrast, the corresponding steady irradiation experiments took place at the natural pH, where the involvement of Cl_2_·^−^ in MA photodegradation is less obvious.

To assess the actual involvement of Cl_2_·^−^ in the steady irradiation process one can check whether $k_{Cl_{2}^{•-}+H_{2}O_{2}}{\left(k_{Cl_{2}^{•-}+MA}\right)}^{-1}$ « kOH+H2O2•(kOH+MA•)−1, as suggested by the steady irradiation results where $R_{MA}$ was directly proportional to ${℘}_{H_{2}O_{2},254nm}^{H_{2}O_{2}+MA}$ (see Figure 2B). Table 2 summarises the second-order reaction rate constants of Cl_2_·^−^, CO_3_·^−^ and ·OH with MA, derived in this study, and those with H_2_O_2_ and HO_2_^−^, obtained from the literature [26,30].

The experimental data of $R_{MA}\;vs.\;[H_{2}O_{2}]$ in the presence of H_2_O_2_ alone (see Figure 2B) are consistent with kOH+H2O2•(kOH+MA•)−1 ~ 0.01. From this value and the condition $k_{Cl_{2}^{•-}+H_{2}O_{2}}{\left(k_{Cl_{2}^{•-}+MA}\right)}^{-1}\;$ « kOH+H2O2•(kOH+MA•)−1, one gets $k_{Cl_{2}^{•-}+MA}$ » 1.4 × 10^7^ M^−1^·s^−1^. The laser flash photolysis experiments yielded $k_{Cl_{2}^{•-}+MA}$ = (4.0 ± 0.3) × 10^8^ M^−1^·s^−1^, in full agreement with the steady irradiation estimate. This means that Cl_2_·^−^ is a reasonable reactive species for the photodegradation of MA in the presence of H_2_O_2_ + Cl^−^ under irradiation in ~neutral solution.

The steady irradiation trend of $R_{MA}\;vs.\;[H_{2}O_{2}]$ in the presence of Na_2_CO_3_, when interpreted in the framework of a competition kinetics between MA and H_2_O_2_ for reaction with CO_3_·^−^, gives kCO3•−+H2O2(kCO3•−+MA)−1>kOH+H2O2•(kOH+MA•)−1 ~ 0.01. From the literature datum $k_{CO_{3}^{•-}+H_{2}O_{2}}$ = 8 × 10^5^ M^−1^·s^−1^ [30] one gets $k_{CO_{3}^{•-}+MA}$ < 8 × 10^7^ M^−1^·s^−1^, which is in stark contrast with the value $k_{CO_{3}^{•-}+MA}$ = (3.1 ± 0.2) × 10^8^ M^−1^·s^−1^ obtained by laser flash photolysis. The H_2_O_2_ + Na_2_CO_3_ solutions had more basic pH (~10) compared to those containing H_2_O_2_ + NaCl, thus H_2_O_2_ would in part occur as its conjugate base HO_2_^−^ [37]. However, when considering that kOH+HO2−• = 7.5 × 10^9^ M^−1^·s^−1^ [26] and $k_{CO_{3}^{•-}+HO_{2}^{-}}$ = 3 × 10^7^ M^−1^·s^−1^ [30], from the condition kCO3•−+HO2−(kCO3•−+MA)−1>kOH+HO2−•(kOH+MA•)−1 one derives $k_{CO_{3}^{•-}+MA}$ < 2 × 10^7^ M^−1^·s^−1^, which is again not consistent with the laser flash photolysis results. A more reasonable explanation is that the reactions of HO_2_^−^ with ·OH and CO_3_·^−^ are much faster than those of H_2_O_2_ (see Table 2), thereby causing a considerable production of HO_2_·/O_2_·^−^ (reactions 11, 12; [26,30]). The superoxide radical anion that prevails at the pH conditions of the studied systems [37] is an effective reductant [38], and it could reduce the oxidised MA transients back to the initial compound (see e.g., reaction 13).

CO_3_·^−^ + HO_2_^−^ → HCO_3_^−^ + O_2_·^−^ (11)

·OH + HO_2_^−^ → H_2_O + O_2_·^−^ (12)

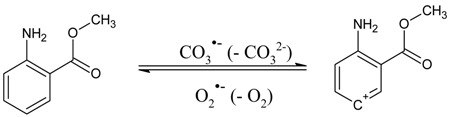
(13)

The above reactions, ending up in an inhibition of MA photodegradation, might explain the trend of $R_{MA}\;vs.\;[H_{2}O_{2}]$ in the presence of carbonate, reported in Figure 2B.

### 2.5. MA Photodegradation Intermediates

**Figure 5:**
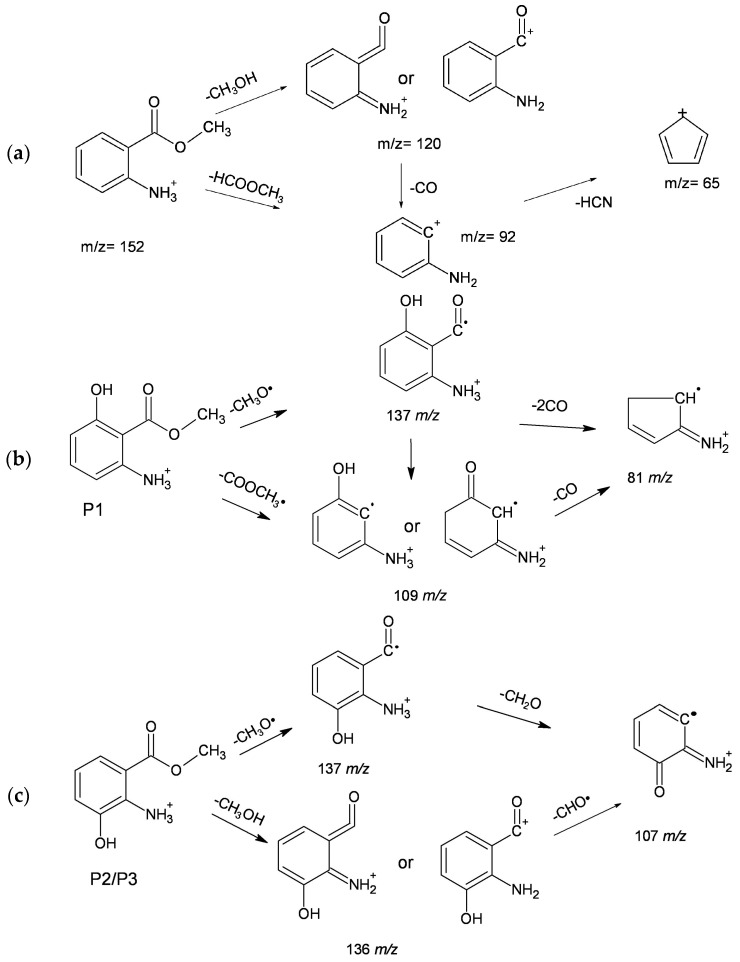
Proposed MS fragmentation pathways of: (**a**) MA; (**b**) a possible structure of P1; (**c**) a possible structure of P2/P3.

The LC-MS analysis of the MA solutions irradiated in the presence of H_2_O_2_, with a conversion percentage of 32%, allowed the detection of MA at the retention time of 12.5 min and of several photodegradation intermediates, namely P1 (10.6 min), P2 (11.0 min), P3 (11.4 min), and P4 (13.1 min). Useful information was initially obtained from the MS spectrum of MA itself. A pattern of MA fragmentation, based on the information obtained in its MS^2^ spectrum at 20 eV, is shown in Figure 5a. The spectrum shows the formation of a fragment ion with an accurate mass of *m/z* = 120.0499, which corresponds to an elemental composition of C_7_H_6_ON^+^ (error = −17 ppm) and is formed by the loss of a CH_3_OH group. This fragmentation is a peculiar behaviour of *ortho*-substituted esters [39]. Two additional fragment ions are also observed at *m/z* 92 and 65. The former with an accurate mass of 92.0500 (C_6_H_6_N^+^, error = −1.3 ppm) arises from the loss of HCO_2_CH_3_ from the molecular ion, which is a common fragmentation process in the methyl esters of carboxylic acids [40]. The same fragment could also be produced by CO loss from the fragment ion at *m/z* 120.0499. The fragment with *m/z* = 65.0391 (C_5_H_5_^+^, error = −3.1 ppm) is obtained from *m/z* = 92.0500 by loss of HCN.

As far as the intermediates P1, P2, and P3 are concerned, they were characterised by the molecular ion *m/z* = 168.0655. This is consistent with the elemental composition C_8_H_10_O_3_N^+^ (error = −3.4 ppm), corresponding to MA monohydroxy derivatives. Remarkably, despite the possibility to hydroxylate MA in four different positions, only three isomers were actually detected with P2 as the major one. The MS^2^ product ions of these compounds are listed in Table 3, together with the LC retention times of the parent molecules.

In the case of P1, the most abundant product ion is 109.0515 *m/z* (C_6_H_7_ON^+^, error = −11.6 ppm), which arises from the loss of CH_3_COO· and is consistent with the presence of the -OH group in position 4 or 6 with respect to the ester functionality of MA. The fragment at 81.0590 *m/z* (C_5_H_7_N^+^, error = +14.2 ppm) can be explained with the further loss of another CO group. The formation of the 141.0569 *m/z* fragment (C_7_H_9_O_3_^+^, error = +12.3 ppm) can be justified with the loss of HCN, whereas the detachment of a CH_3_O· radical group would yield the fragment at 137.0470 *m/z* (C_7_H_7_O_2_N^+^, error = −5.0 ppm). Unfortunately, no further information is present in the spectrum that allows for the determination of the exact location of the OH group. For explanatory purposes, the fragmentation pathway of the 6-hydroxyderivative of MA is reported in Figure 5b. Remarkably, a totally similar fragmentation that yields fragment ions with the same *m/z* values could be proposed for the 4-hydroxyderivative.

As far as P2 and P3 are concerned, the most abundant signal occurs at 136.0375 *m/z* (C_7_H_6_O_2_N^+^, error = −17.3 ppm) and, in analogy with the fragmentation of MA, it could arise from CH_3_OH loss. As already seen for P1, one also observes the product ion at 137.0465 *m/z*. The occurrence of the product ion at 107.0358 *m/z* (H_2_CO loss) suggests the presence of an OH group in *ortho* or *para* position with respect to the amino group (i.e., in position 3 or 5 with respect to the ester functionality). A possible fragmentation pathway for the 3-hydroxyderivative is shown in Figure 5c, but a fully similar pathway could be proposed for the 5-hydroxyderivative. From the available MS data it was unfortunately not possible to attribute uniquely each isomer to the corresponding signal. However, by assuming that P2 and P3 are the 3- and 5-hydroxyderivatives of MA (irrespective of which is which), one can tentatively conclude that both of them are anyway formed. In contrast, P1 may be either the 4- or the 6-hydroxyderivative. Therefore, one could tentatively assume that hydroxylation takes place in the 3 and 5 positions, plus 4 or 6 (in other words, either the 3-, 4-, and 5- or the 3-, 5-, and 6-hydroxyderivatives would be formed).

In the case of P4, the accurate mass of the molecular ion (*m/z* = 331.0915) corresponds to the elemental composition C_16_H_15_N_2_O_6_^+^, with an error of −4.6 ppm. This indicates the possible presence of an oxidised dimeric structure. Unfortunately, based on the available MS data it was not possible to propose a univocal structure for this compound.

Based on ECOSAR predictions, the MA hydroxyderivatives would show comparable toxicity as the parent molecule [7]. In all the cases the major effects are predicted to be the acute and, most notably, the chronic toxicity towards fish and crustaceans.

## 3. Methods

### 3.1. Reagents and Materials

Methyl 2-aminobenzoate (MA, purity grade ≥98%), methanol (gradient grade), HClO_4_ (70%), NaOH (1.0 M titrated solution), and Na_2_CO_3_ (99.9%) were purchased from Sigma-Aldrich (Saint-Quentin-Fallavier, France). Formic acid (98%), H_2_O_2_ (30%), and NaCl (99.5%) were purchased from Fluka (Saint-Quentin-Fallavier, France). The above chemicals were used as received. Ultra-pure water was prepared with a Millipore (Billerica, MA, USA) Milli-Q apparatus (resistivity ≥ 18.2 MΩ cm, TOC < 2 ppb).

### 3.2. Irradiation Experiments

**Figure 6:**
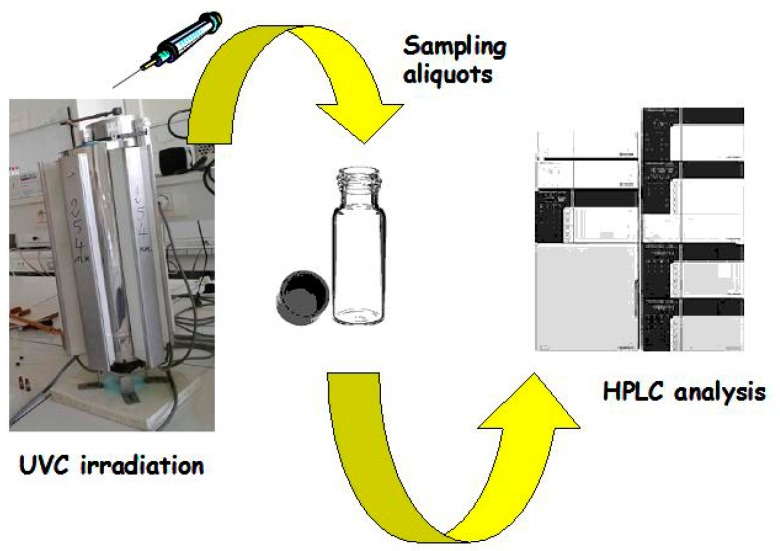
Schematic of the used experimental procedure.

The absorption spectra of the studied compounds (see Figure 1 for MA and H_2_O_2_) were taken with a Varian (Palo Alto, CA, USA) Cary 3 UV-vis spectrophotometer, using 1 cm quartz cuvettes. The solution pH was measured with a combined glass electrode connected to a Meterlab pH meter (Hach Lange, Loveland, CO, USA). Solutions containing 0.1 mM MA, and other components where relevant, were inserted inside a quartz tube (100 mL total volume), which was placed in the centre of an irradiation set-up consisting of six TUV Philips (Amsterdam, Netherlands) 15 W lamps with emission maximum at 254 nm. The lamp intensity was 7.6 × 10^−9^ Einstein cm^−2^·s^−1^. The water solutions were magnetically stirred during irradiation. At scheduled irradiation times, 1.5 mL sample aliquots were withdrawn from the tube, placed into HPLC vials, and kept refrigerated until HPLC analysis. The time trend of MA was monitored by means of a high-performance liquid chromatograph interfaced to a photodiode-array detector (HPLC-PDA, model Nexera XR by Shimadzu, Kyoto, Japan), equipped with SIL20-AC autosampler, SIL-20AD pump module for low-pressure gradients, CT 0-10AS column oven (set at 40 °C), reverse-phase column Kinetex RP-C18 packed with Core Shell particles (100 mm × 2.10 mm × 2.6 µm) by Phenomenex (Torrance, CA, USA), and SPDM 20A photodiode array detector. The isocratic eluent was a A/B = 60/40 mixture of A = (0.5% formic acid in water, pH 2.3) and B = methanol, at a flow rate of 0.2 mL min^−1^. In these conditions, the MA retention time was 7.3 min. The detection wavelength was set at 218 nm. A schematic of the experimental procedure is reported in Figure 6.

The time evolution of MA concentration ([MA]) was fitted with the pseudo-first order equation [MA]_t_ = [MA]_o_ $e^{-k_{MA}\;t}$, where [MA]_t_ is the concentration of MA at the time *t*, [MA]_o_ the initial concentration of MA, and *k*_MA_ the pseudo-first order photodegradation rate constant of MA. The initial rate of MA photodegradation is *R*_MA_ = *k*_MA_ [MA]_o_.

### 3.3. Identification of Photodegradation Intermediates

The photodegradation intermediates of MA were identified by liquid chromatography interfaced with mass spectrometry (LC-MS). A Waters Alliance (Milford, MA, USA) instrument equipped with an electrospray (ESI) interface (used in ESI+ mode) and a Q-TOF mass spectrometer (Micromass, Manchester, UK) were used. Samples were eluted on a column Phenomenex Kinetex C18 (100 mm × 2.10 mm × 2.6 µm) with a mixture of acetonitrile (A) and 0.1% formic acid in water (B) at 0.2 mL·min^−1^ flow rate, with the following gradient: start at 5% A, then up to 95% A in 15 min, keep for 10 min, back to 5% A in 1 min, and keep for 5 min (post-run equilibration). The capillary needle voltage was 3 kV and the source temperature 100 °C. The cone voltage was set to 35 V. Data acquisition was carried out with a Micromass MassLynx 4.1 data system. Both MS and MS/MS experiments were carried out by using this chromatographic set-up.

### 3.4. Laser Flash Photolysis Experiments

The reactivity of the radicals Cl_2_·^−^, CO_3_·^−^, and SO_4_·^−^ was studied by means of the nanosecond laser flash photolysis technique. Flash photolysis runs were carried out using the third harmonic (266 nm) of a Quanta Ray GCR 130-01 Nd:YAG laser system instrument, used in a right-angle geometry with respect to the monitoring light beam. The single pulses energy was set to 35 mJ unless otherwise stated. A 3 mL solution volume was placed in a quartz cuvette (path length of 1 cm) and used for a maximum of three consecutive laser shots. The transient absorbance at the pre-selected wavelength was monitored by a detection system consisting of a pulsed xenon lamp (150 W), monochromator, and a photomultiplier (1P28). A spectrometer control unit was used for synchronising the pulsed light source and programmable shutters with the laser output. The signal from the photomultiplier was digitised by a programmable digital oscilloscope (HP54522A). A 32 bits RISC-processor kinetic spectrometer workstation was used to analyse the digitised signal.

### 3.5. Model Assessment of Toxicity

The potential acute and chronic toxicity of the detected MA intermediates was assessed with the ECOSAR software (US-EPA, Washington DC, USA). ECOSAR uses a quantitative structure-activity relationship approach to predict the toxicity of a molecule of given structure. The relevant endpoints are the acute and chronic toxicity thresholds (LC50, EC50, chronic values ChV) for freshwater fish, daphnid, and algae. The values predicted by ECOSAR are apparently very precise but, as far as accuracy is concerned, a compound can be said to be more toxic than another only when the predicted values differ by at least an order of magnitude [7,8].

## 4. Conclusions

The H_2_O_2_/UV technique as photochemical ·OH source is a potentially effective tool to achieve MA photodegradation, and in fact the addition of hydrogen peroxide considerably accelerated the photodegradation of MA compared to UV irradiation alone. The addition of inorganic anions that act as ·OH scavengers, such as chloride and carbonate, did not necessarily quench MA photodegradation. The reason is the reactivity with MA itself of the generated radical species, i.e., Cl_2_·^−^ produced from Cl^−^+·OH and CO_3_·^−^ produced from CO_3_^2−^+·OH. In the case of chloride, there was even an acceleration of MA photodegradation at elevated [H_2_O_2_], because Cl_2_·^−^ competes more successfully than ·OH for reaction with MA in the presence of H_2_O_2_ (H_2_O_2_ behaves as a scavenger of ·OH and, to a lesser extent, of Cl_2_·^−^ as well). The same effect was not observed with carbonate, possibly because the basic pH caused a considerable production of superoxide (O_2_·^−^) upon oxidation of the H_2_O_2_ conjugated base, HO_2_^−^. The radical O_2_·^−^ is a well-known reductant that could reduce the partially oxidised MA back to the starting compound. Effective MA photodegradation was also observed with persulphate/UV, probably because of the fast reaction between MA and photogenerated SO_4_·^−^, and because of limited scavenging of SO_4_·^−^ by persulphate itself.

Among the MA photodegradation intermediates detected in the H_2_O_2_/UV process, the hydroxyderivatives could be about as toxic as the parent compound. Therefore, decontamination is not yet achieved once MA has disappeared, and the H_2_O_2_/UV treatment of MA should at least ensure the photodegradation of the MA hydroxylated derivatives as well. Usually, the photodegradation of both the primary compound and its intermediates takes more time than the photodegradation of the starting compound alone.

## Figures and Tables

**Table 1:** Pseudo-first order photodegradation rate constants of MA (*k*_MA_) in the different irradiation experiments. The initial concentration values of hydrogen peroxide, persulphate, chloride and carbonate are also reported. The error bounds to the *k*_MA_ data represent the sigma-level uncertainty of the pseudo-first order kinetic model. In all the cases the initial concentration of MA was 0.1 mM.

[H_2_O_2_], mM	[S_2_O_8_^2−^], mM	[Cl^−^], mM	[CO_3_^2−^], mM	*k*_MA_, min^−1^ (±σ)
0	/	/	/	0.081 ± 0.007
5	/	/	/	2.72 ± 0.12
10	/	/	/	2.03 ± 0.13
20	/	/	/	2.87 ± 0.09
5	/	100	/	2.29 ± 0.16
10	/	100	/	2.97 ± 0.06
20	/	100	/	5.30 ± 0.34
5	/	/	100	1.40 ± 0.08
10	/	/	100	0.652 ± 0.121
20	/	/	100	0.764 ± 0.049
/	0	/	/	0.106 ± 0.007
/	1	/	/	0.133 ± 0.031
/	5	/	/	0.598 ± 0.018
/	10	/	/	7.09 ± 0.16

**Table 2:** Second-order reaction rate constants (*k*_X+Y_) between the transient species X = Cl_2_·^−^, CO_3_·^−^ or ·OH, and the compound Y = MA, H_2_O_2_, or HO_2_^−^.

*k*_X+Y_, M^−1^ s^−1^	MA	H_2_O_2_	HO_2_^−^
**Cl_2_·^−^**	4.0 × 10^8^	1.4 × 10^5^ [30]	n/a
**CO_3_·^−^**	3.1 × 10^8^	8 × 10^5^ [30]	3 × 10^7^ [30]
**·OH**	~10^9^	2.7 × 10^7^ [26]	7.5 × 10^9^ [26]

**Table 3:** Summary of the mass spectrometric data of the detected MA hydroxyderivatives.

Compound Acronym	LC Retention Time, (min)	MS^2^ Fragments, *m/z* (% Relative Abundance)
**P1**	10.6	141 (8), 137 (7), 109 (100), 81 (38)
**P2**	11.0	136 (100), 137 (30), 107 (3)
**P3**	11.4	136 (100), 137 (25), 107 (25)

## Supplementary Materials

Supplementary materials are available online.

## Abbreviations

*k*_MA_	pseudo-first order rate constant of MA photodegradation.
*k*_X+Y_	second-order reaction rate constant between the species X and the compound Y.
*R*_MA_	initial photodegradation rate of MA.
ε_Y,λ_	molar absorption coefficient of the compound Y at the wavelength λ.
*p*°(λ)	incident spectral photon flux density of the used lamp at the wavelength λ.
$R_{X}^{Y+h\nu }$	formation rate of the species X in the presence of the compound Y under irradiation.
${℘}_{Y,\lambda }^{S}$	fraction of radiation absorbed by the compound Y at the wavelength λ, inside the solution S.
