# Supplementary material for: Degradation of Methyl 2-Aminobenzoate (Methyl Anthranilate) by H2O2/UV: Effect of Inorganic Anions and Derived Radicals
Source: Molecules. 2017 Apr 12;22(4):619. doi: 10.3390/molecules22040619 (PMC6154702; doi:10.3390/molecules22040619)
Supplement: Supplementary file 1 [file molecules-22-00619-s001.pdf]

SUPPLEMENTARY MATERIAL

DEGRADATION OF METHYL 2-AMINOBENZOATE (METHYL ANTHRANILATE) BY H_2O_2/UV: EFFECT OF INORGANIC ANIONS AND DERIVED RADICALS

Grazia Maria Lanzafame,[1,2,3] Mohamed Sarakha,[1] Debora Fabbri,[2] Davide Vione,[2,4]*

1. *Clermont Université, Université Blaise Pascal, Institut de Chimie de Clermont-Ferrand, F-63177 Aubière, France.*
2. *Dipartimento di Chimica, Università di Torino, Via Pietro Giuria 5, 10125 Torino, Italy.* http://www.chimicadellambiente.unito.it
3. *Present address: INERIS, Rue Jacques Taffanel, F-60550 Verneuil-en-Halatte, France.*
4. *Università di Torino, Centro Interdipartimentale NatRisk, Largo Paolo Braccini 2, 10095 Grugliasco (TO), Italy.* http://www.natrisk.org

Direct photolysis quantum yield of methyl anthranilate (MA)

The value of the direct photolysis quantum yield (Φ_{MA}) was measured upon monochromatic and polychromatic irradiation of 0.2 mM MA solutions placed inside spectrophotometer cuvettes. The initial MA concentration was chosen to obtain absorbance values in the range of 0.5 to 0.7 at the studied wavelengths. Monochromatic radiation at 254 and 325 nm was obtained by a suitable combination of Horiba xenon lamps and diffraction-grid monochromators, and irradiation was carried out till 15% disappearance of the initial MA. Polychromatic irradiation at λ > 220 nm was obtained by using an Atlas Suntest® apparatus (unfiltered xenon lamp emission). The spectral photon flux density emitted by the radiation sources was measured with a calibrated Ocean Optics USB 2000 CCD camera.

The photolysis quantum yield Φ_{MA} was obtained as follows:

$$\Phi_{MA} = \frac{R_{MA}}{2.303\, b[MA] \int_{\lambda} p°(\lambda)\ \varepsilon_{MA}(\lambda)\, d\lambda} \quad \text{(SM1)}$$

where R_{MA} is the initial photodegradation rate of MA (initial concentration [MA] = 0.2 mM), b = 1 cm the optical path length of the cuvette, $\varepsilon_{MA}(\lambda)$ the molar absorption coefficient of MA, and $p°(\lambda)$ the measured spectral photon flux density emitted by the radiation source. In the case of monochromatic irradiation at a given wavelength (assuming $\Delta\lambda$ = 1 nm) it is $\int_{\lambda} p°(\lambda)\ \varepsilon_{MA}(\lambda)\, d\lambda = p°(\lambda)\ \varepsilon_{MA}(\lambda)$.

The following Table reports the reaction parameters as well as the photochemical data that were used to determine Φ_{MA}.

Table SM1. Data used for the calculation of $\mathbf{\Phi_{MA}}$ under the different irradiation conditions. n/a = not applicable.

Irradiation conditions	R_{MA}, mol L^{-1} s^{-1}	$A_{MA}(\lambda) = \varepsilon_{MA}(\lambda)$ b [MA]	$\int_{\lambda} p°(\lambda)\ \varepsilon_{MA}(\lambda)\, d\lambda$, ein L^{-1} s^{-1}	$\int_{\lambda} p°(\lambda)\, d\lambda$, ein L^{-1} s^{-1}	Φ_{MA}
254 nm	$6.0 \cdot 10^{-10}$	0.50	n/a	$1.4 \cdot 10^{-6}$	$3.8 \cdot 10^{-3}$
325 nm	$1.4 \cdot 10^{-9}$	0.53	n/a	$2.3 \cdot 10^{-7}$	$1.4 \cdot 10^{-3}$
Suntest	$1.8 \cdot 10^{-8}$	n/a	$2.33 \cdot 10^{-2}$	n/a	$1.7 \cdot 10^{-3}$
